# The number and type of food retailers surrounding schools and their association with lunchtime eating behaviours in students

**DOI:** 10.1186/1479-5868-10-19

**Published:** 2013-02-07

**Authors:** Laura Seliske, William Pickett, Andrei Rosu, Ian Janssen

**Affiliations:** 1 Department of Community Health & Epidemiology, Queen’s University, Kingston, ON, K7L 3N6, Canada; 2 Department of Emergency Medicine, Kingston General Hospital, Kingston, ON, K7L 2V7, Canada; 3 School of Kinesiology and Health Studies, Queen’s University, 28 Division St, Kingston, ON, K7L 3N6, Canada

**Keywords:** Eating behaviours, Youth, Food retail environment, Schools

## Abstract

**Background:**

The primary study objective was to examine whether the presence of food retailers surrounding schools was associated with students’ lunchtime eating behaviours. The secondary objective was to determine whether measures of the food retail environment around schools captured using road network or circular buffers were more strongly related to eating behaviours while at school.

**Methods:**

Grade 9 and 10 students (N=6,971) who participated in the 2009/10 Canadian Health Behaviour in School Aged Children Survey were included in this study. The outcome was determined by students’ self-reports of where they typically ate their lunch during school days. Circular and road network-based buffers were created for a 1 km distance surrounding 158 schools participating in the HBSC. The addresses of fast food restaurants, convenience stores and coffee/donut shops were mapped within the buffers. Multilevel logistic regression was used to determine whether there was a relationship between the presence of food retailers near schools and students regularly eating their lunch at a fast food restaurant, snack-bar or café. The Akaike Information Criteria (AIC) value, a measure of goodness-of-fit, was used to determine the optimal buffer type.

**Results:**

For the 1 km circular buffers, students with 1–2 (OR= 1.10, 95% CI: 0.57-2.11), 3–4 (OR=1.45, 95% CI: 0.75-2.82) and ≥5 nearby food retailers (OR=2.94, 95% CI: 1.71-5.09) were more likely to eat lunch at a food retailer compared to students with no nearby food retailers. The relationships were slightly stronger when assessed via 1 km road network buffers, with a greater likelihood of eating at a food retailer for 1–2 (OR=1.20, 95% CI:0.74-1.95), 3–4 (OR=3.19, 95% CI: 1.66-6.13) and ≥5 nearby food retailers (OR=3.54, 95% CI: 2.08-6.02). Road network buffers appeared to provide a better measure of the food retail environment, as indicated by a lower AIC value (3332 vs. 3346).

**Conclusions:**

There was a strong relationship between the presence of food retailers near schools and students’ lunchtime eating behaviours. Results from the goodness of fit analysis suggests that road network buffers provide a more optimal measure of school neighbourhood food environments relative to circular buffers.

## Background

Poor eating behaviours, defined in this paper as eating behaviours that lead to an increased consumption of foods high in calories, sugar, salt and fat, are an important determinant of health among young people. Consumption of these foods is associated with the onset of several adverse health outcomes including obesity [1-3] and early indicators of cardiovascular disease [4,5] and type 2 diabetes [6]. Young people who frequently eat at fast food restaurants have poorer diets than those who eat at these restaurants less frequently [7-9]. Although there is a lack of analogous research on food purchases at convenience stores or coffee/donut shops, the increased consumption of sugar sweetened beverages [10] and snack foods [11] in recent years suggests that these food retailers may also influence eating behaviours.

Since young people spend a large portion of their day at school, the school food environment may impact their eating behaviours and diets. Most research on the school food environment has focused directly on the school itself (e.g., cafeterias, vending machines) [12-14]. However, many students are permitted to leave school grounds during the school day and have access to nearby food retailers. There is sometimes a preponderance of fast food restaurants near schools [15,16], and these types of food retailers sell primarily unhealthy foods. A few studies have considered whether the presence of food retailers near schools negatively influences young peoples’ eating behaviours and diets. Findings from these studies provided weak [17] to no support of this notion [18,19]. A major limitation of existing studies is that they measured the overall consumption of specific food items (e.g., fruits and vegetables), and did not consider where or when the food items were obtained. This makes it impossible to distinguish between the contribution of the school food retail environment, the home environment, and other environments to overall consumption. There is a need for studies to examine how the presence of food retailers, both within schools and in the surrounding area, influences students’ eating behaviours during the school day. It is also important to note that although young people spend a large portion of their day at school, there are other locations such as the home food environment where context-specific eating behaviours are also important.

In order to measure the local food retail environment, previous studies have used different types of geographic boundaries, with most relying on either circular buffers [17,19] or road network buffers [15,18]. Circular buffers capture all land within a set distance from a location of interest “as the crow flies”, while road network buffers extend outwards from a location of interest by following road networks, and therefore capture what is actually accessible to a person by road. Circular buffers, while easy to create, do not necessarily reflect how people travel. Road network-based buffers address this limitation, but the creation of road network buffers requires more time and expertise in geographic information system (GIS) technologies. Furthermore, students who walk to food retailers near their school may take pathways and short cuts which would not be captured by the road network buffers. Measures of the built environment captured using road network buffers have been shown to be more strongly and consistently related to physical activity behaviours than circular buffers measures in adults [20]. There is an analogous need to address this buffer measurement issue for the food retail environment.

The primary objective of this study was to examine the relationship between the food retail environment surrounding Canadian schools and students’ lunchtime eating behaviours, a relationship that has not been demonstrated convincingly in past studies potentially due to a lack of precision in the measurement of specific eating behaviours and locations. A secondary objective was to determine whether measures of the food environment obtained by road network buffers were more strongly related to eating behaviours than comparable measures obtained using circular buffers. We had the opportunity to study these objectives in a large national study. From a public health perspective, findings from this study could support strategies aimed at improving eating behaviours among young people through the development of policies that would address the food retail environment surrounding schools. From a methodological perspective, the findings of this study could provide information on the optimal buffer type to use when performing research on the food retail environment.

## Methods

### Overview of study design

This study involved a multilevel cross-sectional analysis that examined the relationship between the food environment surrounding schools and the locations where grade 9 and 10 students ate their lunch during the school week. Participants and schools were obtained from the 2009/10 Canadian Health Behaviour in School-Aged Children (HBSC) survey. Addresses of food retailers surrounding schools were gathered using an online food retailer database. Presence of food retailers surrounding each school was measured by creating 1 km circular and 1 km road network buffers around schools using the GIS software, then determining the number of food retailers within each type of buffer. Associations between food retailer exposures and individual reports from students about eating their lunch at a food retailer were then assessed.

### Study sample

The HBSC survey is carried out in association with the World Health Organization and was conducted in 43 countries in 2009/10. It includes a student survey completed by grades 6 to 10 students and an administrator survey completed by a principal or designate at each of the participating schools. The student survey is completed in the classroom and covers a variety of health behaviours and their determinants in students in grades 6 to 10 (approximate ages of 11 to 16 years). In Canada, a single stage cluster sampling approach was used to obtain participants, in accordance with an international protocol [21]. Classes were the primary sampling unit, and they were stratified by province, with an oversampling of some provinces and the three northern territories. The HBSC excludes students in private schools, incarcerated youth, special needs schools and students who are home schooled. In 2010, two Canadian provinces with small populations (New Brunswick and Prince Edward Island) were unable to participate. Ethics approval was obtained from the Queen’s University General Research Ethics Board. Subject consent was obtained at the school board and school levels as well as from parents or guardians (either explicitly or implicitly, as determined by school board policy).

The 2009/10 HBSC contained a total of 26,078 students in grades 6 to 10 who attended 436 schools across Canada. The analyses were restricted to students who attended schools that allowed students to leave school grounds during the school day (e.g., during their lunch), thereby making it possible for them to purchase food at nearby food retailers. The sample was limited to students in grades 9 and 10 because only a small proportion (1.1%) of students in grades 6 to 8 ate their lunch at a food retailer. An additional 11 schools were removed from the study sample because information was not available on food sources within the school or in the school neighbourhood. Finally, 940 students were excluded because of missing data on key variables. The final analyses involved 6,971 students from 158 schools.

### Eating lunch at a food retailer

The outcome of this study was obtained from the response to the following question: “*Where do you usually eat your lunch or mid-day meal on school days?”* Students who chose the response *“snack-bar, fast food restaurant, café”* were classified as regularly purchasing their lunch from a food retailer. Those who chose the remaining responses (*“at school”, “at home”, “at someone else’s house”, “do not eat lunch/mid-day meal”,* or *“other”*) were classified as not having that behaviour.

### School food retail environment

**Figure 1:**
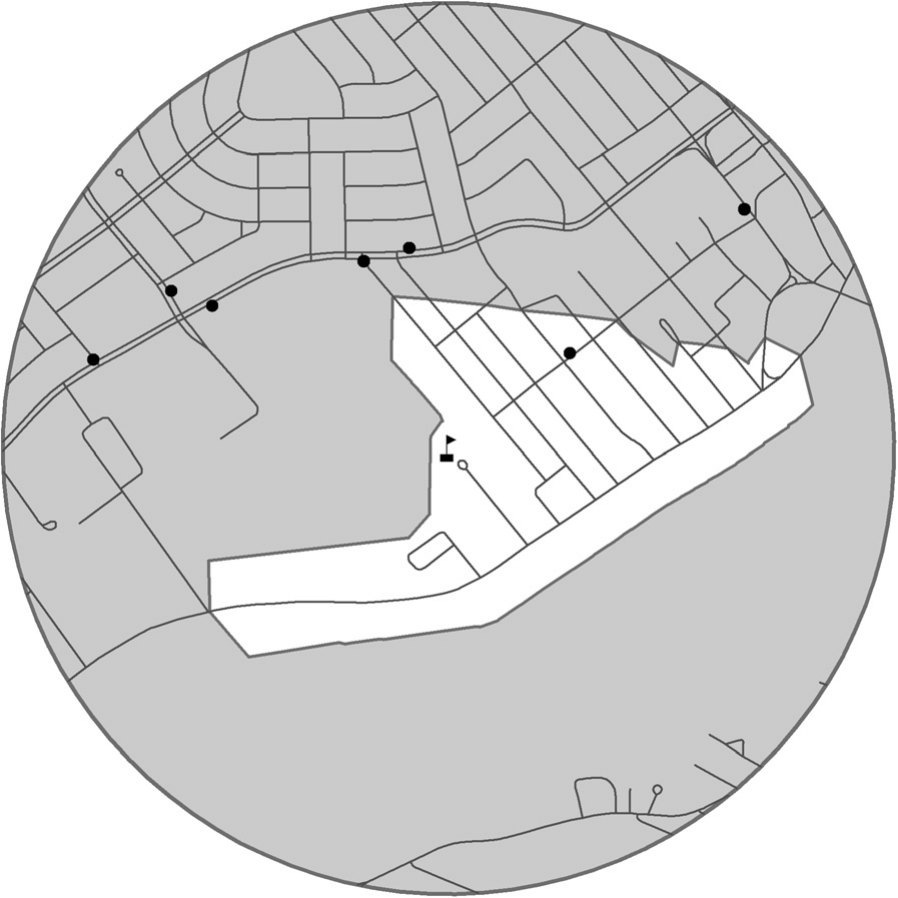
**Comparison of 1 km circular and road network buffers to measure access to food retailers for one school.** The road network buffer is represented by the white area and the circular buffer encompasses both the white and gray areas. The school is represented by the flag in the centre of the circle and the food retailers are represented by the black circles.

The addresses of the 158 HBSC schools were mapped in ArcGIS (ESRI, version 9.3) and 1 km circular and road network-based buffers were constructed. The 1 km distance was chosen because it approximates the distance that can be walked in 10 to 15 minutes [22], a comfortable amount of time for students to walk to and from food retailers during their lunch break. Circular buffers surrounding schools were created by extending a 1 km radius around the schools. Road network buffers were created using a commercial road network database provided by CanMaps Streetfiles database (DMTI Spatial Inc., v.2009.4, Markham ON). Roads extending outwards from schools were followed until they reached an endpoint at 1 km. Lines connecting the 1 km endpoints were used to create the border of the road network-based buffers. To illustrate, the Figure 1 shows the characteristics of a circular and road network buffer for one of the participating HBSC schools. For some schools, the 1 km road network buffer may cover a considerably smaller area than circular buffers and therefore capture fewer food retailers.

Convenience stores, fast food restaurants, and coffee/donut shops were included as the primary independent variable of interest. These directly corresponded to the lunchtime eating question used to infer the study outcome. Information on the addresses of these types of food retailers near schools was obtained using an online Yellow Pages directory (http://www.yellowpages.ca). The Yellow Pages database was chosen because it provided more accurate information on food retailer locations than a commercial database [23]. School addresses were entered into the Yellow Pages directory. The search term ‘convenience stores’ was used to obtain convenience stores. There was no single search term to use for fast food restaurants and coffee/donut shops because many of them were listed under the full service restaurant category. Therefore, we searched for the top 75% of the top 200 chain food retailers for Canada in 2009 [24], similar to what has been done in previous studies [25,26]. Names of the 16 fast food restaurant chains and 4 coffee/donut chain restaurants that were included as search terms are shown in Table 1.

**Table 1:** Top 75% of Canadian chain fast food retailers and coffee/donut shops by number of outlets

**Fast food retailers**	**Number of outlets**	**Coffee/donut shops**	**Number of outlets**
Subway	2477	Tim Hortons	3014
McDonald’s	1420	Starbucks	1051
KFC	760	Country Style	465
A&W	700	Second Cup	343
Pizza Pizza	590		
Dairy Queen	580		
Quizno’s	450		
Mr Sub	400		
Burger King	395		
Wendy’s	371		
Pizza Hut	340		
Domino’s Pizza	319		
Harvey’s	260		
Little Caesar’s	191		
New York Fries	180		
Taco Bell	175		

All food retailers within the 1 km circular and 1 km road network buffers were mapped using ArcGIS software. For food retailers whose street addresses had a match score of less than 80%, the Street View tool in Google Earth (©2011 Google) was used to confirm the location and obtain latitude and longitude coordinates to map them manually in ArcGIS. The number of food retailers within the buffers was positively skewed, hence the following categories were created: no food retailers present, presence of 1 or 2 food retailers, presence of 3 or 4 food retailers, and presence of 5 or more food retailers.

### Confounders

Individual-level variables, including age, sex, and socioeconomic status were considered as potential confounders since fast food consumption varies by these characteristics [9,27,28]. To obtain information on socioeconomic status, the HBSC uses the previously validated family affluence scale (FAS) [29]. Finally, because cafeterias, vending machines, and school snack/tuck shops are associated with students’ eating behaviors [12-14], they were considered as potential school-level confounders as they may have reduced the likelihood of students seeking food from food retailers outside of the school.

### Analysis

All analyses were conducted using SAS statistical software, version 9.2 (SAS Institute, Cary, NC). Multilevel logistic regression was carried out to examine the relationship between the presence of food retailers (convenience stores, fast food restaurants, and coffee/donut shops) and the likelihood of students eating their lunch at these food retailers. A three step modeling procedure was carried out. First, an empty model was used to determine the intra-class correlation (ICC) statistic; the latter provides an estimate of the proportion of the variation in the study outcome that was due to differences between schools. Second, bivariate relationships were examined between the study outcome and each potential confounder. Finally, the multivariate model building process began with the introduction of the individual-level confounders and proceeded using a backwards elimination approach. Next, the school level food exposure variables were forced into the model because we were interested in assessing food sources within school as well as those surrounding schools.

The Akaike information criterion (AIC), which is a measure of goodness-of-fit when comparing two or more regression models, was determined for the final multivariate models. A difference in AIC scores between 2 to 7 indicates a moderate difference in fit of the models, while a difference of 7 or more indicates a large difference in model fit [30]. Using the AIC values, the Akaike weights were calculated, and they indicate the probability that a regression model is the best choice among a set of candidate models based on the model fit [31]. After the model with the best fit was determined, the population attributable risk (PAR) was calculated, using the following formula: PAR = P_exp_(RR - 1)/1 + P_exp_(RR - 1), where P_exp_ denotes the prevalence of the exposure [32] and RR denotes the relative risk. Since the outcome is rare (<10%), the odds ratio (OR) was used to approximate the RR.

## Results

### Description of the study sample

Table 2 shows the individual-level characteristics of the study sample. There was an approximately equal distribution of males and females. Over half of the participants were in the highest family affluence group and only 8.0% were in the lowest. Of the participants who provided self-reported weight and height, 19.6% were overweight or obese according to the International Obesity Task Force body mass index criteria [33]. During the school week, the majority of students typically ate their lunch either at school (67.7%), at home (15.2%), or in a snack-bar, fast food restaurant, or café (7.4%).

**Table 2:** Characteristics of the study sample from the 2009/10 Health Behaviour in School Aged Children Survey

	**N**	**%**
**Sex**		
Male	3381	48.5
Female	3590	51.5
**Age (years)**		
13	33	0.5
14	2339	33.6
15	3280	47.1
16	1319	18.9
**Family affluence scale**		
Low	560	8.0
Moderate	2531	36.3
High	3880	55.7
**Weight status**		
Non-overweight	4823	69.2
Overweight	1018	14.6
Obese	346	5.0
Missing data	784	11.3
**Where students eat mid-day meal**		
At school	4719	67.7
At home	1056	15.2
In a snack-bar, fast food restaurant or café	517	7.4
Never eat a midday meal	307	4.4
Somewhere else	209	3.0
At someone else’s home	163	2.3

Characteristics of the schools involved in this analysis are shown in Table 3. The majority were secondary schools (limited to students in grades 9 to 12) and 39.2% were located within large urban centres. Most schools had cafeterias (76.0%) and vending machines that served sugared drinks (61.4%). Only 32.3% of schools had a snack/tuck shop. Overall, 70.9% of schools had at least one food retailer of any type within the circular buffer, and 61.3% of schools had at least one food retailer within the road network buffer. Convenience stores and fast food restaurants were most prevalent. A total of 648 food retailers were located within the 1 km circular buffers surrounding the 158 schools, and 394 food retailers were located within the 1 km road network buffers.

**Table 3:** Characteristics of the school sample from the 2009/10 Health Behaviour in School Aged Children Survey

	**N**	**%**
**School type**		
Secondary (grades 9–12)	94	59.5
Mixed	64	40.5
**Urban rural status**		
Large urban centre (≥100,000 people)	62	39.2
Medium urban centre (20,000 - 99,999 people)	15	9.5
Small urban centre (1,000 - 19,999 people)	38	24.1
Rural (<1,000 people)	43	27.2
**Access to food sources within school**		
Cafeteria	120	76.0
Sugared drinks vending machines	97	61.4
Milk vending machines	75	47.5
Candy and potato chip vending machines	64	40.5
School tuck shop/snack-bar	51	32.3
**≥1 Food retailer within 1 km circular buffer**		
Convenience stores	92	55.2
Fast food restaurants	88	55.7
Coffee/donut shops	53	33.5
All food retailers	112	70.9
**≥1 Food retailer within 1 km road network buffer**		
Convenience stores	73	46.2
Fast food restaurants	65	41.1
Coffee/donut shops	35	22.2
All food retailers	103	61.3

### Association between neighborhood food environments and lunchtime eating behaviours

The ICC value indicated that 26.3% of the variation in the lunchtime eating outcome was due to school-level factors. The high ICC value provides support for the need to use multi-level modeling to examine the relationship between the presence of food retailers near schools, measured by 1 km circular and 1 km road network buffers, and the lunchtime eating outcome (Table 4). The bivariate analysis showed that the only individual-level confounder related to lunchtime eating was sex, with males nearly twice as likely to obtain their lunch from a food retailer. For the school-level confounders, the presence of a school snack/tuck shop decreased the likelihood of eating at food retailers by nearly half while the presence of a cafeteria in the school was positively related to students eating lunch at a food retailer.

**Table 4:** Food retailers surrounding schools and eating lunch at a café, fast food restaurant or snack-bar

	***Bivariate***		***Individual level variables***		***Individual and school level variables***	
	**Circular buffer**	**Network buffer**	**Circular buffer**	**Network buffer**	**Circular buffer**	**Network buffer**
***Number of food retailers***						
0	1.00	1.00	1.00	1.00	1.00	1.00
1-2	1.08 (0.57-2.04)	1.24 (0.76-2.02)	1.08 (0.57-2.06)	1.25 (0.76-2.06)	1.10 (0.57-2.11)	1.20 (0.74-1.95)
3-4	1.45 (0.76-2.76)	3.30 (1.71-6.37)	1.45 (0.76-2.79)	3.27 (1.67-6.39)	1.45 (0.75-2.82)	3.19 (1.66-6.13)
≥ 5	3.00 (1.77-5.09)	3.59 (2.13-6.05)	3.08 (1.80-5.27)	3.70 (2.17-6.30)	2.94 (1.71-5.09)	3.54 (2.08-6.02)
***Individual-level variables***						
**Age**						
1 year increase	1.09 (0.94-1.26)	1.09 (0.94-1.26)				
**Sex**						
Female	1.00	1.00	1.00	1.00	1.00	1.00
Male	1.91 (1.57-2.33)	1.91 (1.57-2.33)	1.92 (1.57-2.33)	1.90 (1.56-2.32)	1.92 (1.57-2.33)	1.91 (1.57-2.32)
**Family affluence scale**						
Low wealth	1.00	1.00				
Medium wealth	0.99 (0.69-1.42)	0.99 (0.69-1.42)				
High wealth	1.00 (0.70-1.42)	1.00 (0.70-1.42)				
***School-level variables***						
**Food sources in schools**						
Cafeteria	1.79 (1.01-3.15)	1.79 (1.01-3.15)	--	--	1.30 (0.75-2.28)	1.49 (0.88-2.53)
Sugared drinks vending	1.20 (0.76-1.91)	1.20 (0.76-1.91)	--	--	1.36 (0.80-2.32)	1.37 (0.82-2.28)
Milk vending	1.15 (0.74-1.77)	1.15 (0.74-1.77)	--	--	1.19 (0.77-1.83)	1.22 (0.81-1.84)
Candy/potato chip vending	1.13 (0.72-1.76)	1.13 (0.72-1.76)	--	--	0.76 (0.44-1.30)	0.76 (0.45-1.26)
School tuck shop/snack-bar	0.57 (0.36-0.91)	0.57 (0.36-0.91)	--	--	0.66 (0.42-1.03)	0.68 (0.44-1.05)

Note: Data are presented as odds ratios (95% confidence interval).

After adjusting for the relevant individual- and school-level confounders, students exposed to ≥5 food retailers based upon the 1 km circular buffer were 2.94 (95% CI: 1.71-5.09) times more likely to eat their lunch at a food retailer compared to students with no food retailers surrounding their school. For the road network-based buffers, students exposed to 3 or 4 food retailers were 3.19 (95% CI: 1.66-6.13) times more likely to eat their lunch at a food retailer and students exposed to ≥5 food retailers were 3.54 (95% CI: 2.08-6.02) times more likely to eat their lunch at a food retailer compared to students with no food retailers surrounding their school.

### Circular vs. Road network buffers

The AIC criterion for the final road network buffer model was 14 units smaller than the value for the final circular buffer model (3332 vs. 3346), indicating that the road network-based buffers provided a better model fit. In addition, the Akaike weight for the road network buffer was 0.99, indicating that there was a 99% probability that the road network data provided the better model fit.

## Discussion

The key findings of our study are as follows. First, by using a focused measure of where students eat their lunch, we were able to demonstrate that the food retail environment surrounding schools is strongly related to student’s eating behaviours during the school day. Second, our findings suggest that the geographic boundaries used to assess the food retail environment are better captured using road network buffers rather than circular buffers.

At 26.3%, the amount of variation in students’ lunchtime eating behaviours attributable to school-level factors was noteworthy. Although there are currently no studies with a similar lunchtime eating behaviour outcome, a similar Canadian study of the school food environment and obesity by Leatherdale et al. [34] had an ICC of 5.4%. Our comparatively higher ICC value indicates that the school food environment accounts for a notable proportion of the variation in students’ lunchtime eating behaviours. Furthermore, the relationship between food retailers surrounding schools and students’ eating behaviors observed in our study were much stronger than those previously reported [17,18]. This difference may partly be explained by our use of a precise measurement of food consumption at food retailers during the school day, rather than a more general measure of food consumption patterns reported for the entire day or week used in past studies [17,18]. It is important to consider the specific context of food consumption, including where and when the food was eaten, in order to evaluate the importance of a specific food environment. By accounting for the particular context in which food was consumed, the potential for the misclassification in the measurement of the food environment is greatly reduced.

Our comparison of the model fit provided by circular and road network-based buffers provided findings consistent with similar research, despite the fact that previous research has used different behavioural outcomes in varying populations. Within a sample of adults residing in Vancouver, B.C. Oliver et al. [20] examined the relationship between land use mix in the 1 km surrounding each participant’s home, measured using 1 km circular and road network buffers, and their walking behaviour. The road network-based measures were consistently related to walking behaviours while the circular-based measures were not. Taken together, the findings from these two studies suggest that researchers should consider investing the time in obtaining road network buffers when they want to measure the association between built environment constructs and health-related behaviours. However, it is important to note the scarcity of studies directly comparing buffer types. Future studies comparing these measures are needed to confirm this observation, particularly when assessing the food environment.

Despite the implementation of a new policy restricting fast food restaurants in a socioeconomically disadvantaged area in California [35], there is currently no evidence evaluating its effectiveness. In fact, to our knowledge, no existing studies have examined whether policies aimed at restricting the number of food retailers that sell primarily unhealthy foods (e.g., fast food restaurants) in a given region impacts people’s dietary behaviours. However, there is analogous evidence from interventions and policies put in place to address the lack of supermarkets – the main source of reasonably priced fresh fruits and vegetables – in socioeconomically disadvantaged neighbourhoods. For instance, the introduction of a new supermarket in a deprived neighborhood in Leeds, UK positively impacted the fruit and vegetable consumption of the adults residing in that neighborhood, particularly those with the worst diets, whose fresh fruit and vegetable consumption doubled [36]. This demonstrates the potential to influence peoples’ eating behaviours by modifying their food environment through policies and interventions. Given the preponderance of unhealthful food retailers near schools [15,16] and the strong associations we found with students’ eating behaviours, there is a need for future research to evaluate whether restrictions on food retailers near schools affect lunchtime eating behaviours of young people.

While the food retail environment within the school has an important impact on student’s eating behaviors and food choices [12-14], approximately one third of the grade 9 and 10 students in our national study did not usually eat their lunch at school and almost one in ten usually ate their lunch at a food retailer. Interestingly, we observed that the presence of school cafeterias and certain vending machines were positively associated, albeit not statistically significant, with eating lunch at food retailers. The positive relationships suggest that despite having the option to purchase food directly within their school, some students prefer to purchase their lunch at nearby food retailers. Furthermore, the population attributable risk calculations suggest that 58% of the study outcome (eating at food retailers during the school week) was attributable to students being exposed to 3 or more food retailers within a 1 km travel distance of their school. Therefore, policies and programs directed at eliminating unhealthy food choices within school cafeterias and vending machines may be undermined by the availability of less nutritious food at nearby food retailers.

If additional studies provide evidence of a strong and consistent relationship between the food environment surrounding schools and students’ eating behaviours, municipal planners should consider implementing policies that would limit the number of food retailers in close proximity to schools. A second strategy to limit students’ consumption of food from nearby food retailers would be the implementation of policies preventing students from leaving school grounds during the school day. However, this would not be effective in preventing students from purchasing food at nearby food retailers before or after school.

Some important strengths and limitations merit consideration. Key strengths of this study included our specific measurement of eating behaviours during the school day and the large and geographically diverse study sample. A limitation of this study was that the food environment was measured with an online GIS database, which may not provide a completely accurate and up-to-date measure of the food environment. Furthermore, only the top 75% of chain fast food and donut/coffee retailers were included in the exposure measure. There was no information from the HBSC survey on which food retailers the students actually went to. Also, all data were obtained by self-report, and may be subject to bias introduced by the social desirability of eating healthy meals. Furthermore, the HBSC survey did not collect information on food preparation practices at home which may influence whether students brought their lunch from home or purchased their lunch from a nearby food retailer. Finally, the study was cross-sectional and therefore temporality between the presence of food retailers and eating behaviours cannot be directly established. However, it is unlikely that students chose to attend schools based on the presence of nearby food retailers.

## Conclusions

In conclusion, this national study provides novel insights into the relationship between the food retail environment surrounding schools and the eating behaviours of students during the school day. It also provided evidence to support the use of network buffers over circular buffers when assessing the food retail environment. Future research needs to evaluate whether policies directed at modifying the food retail environment improve eating behaviours.

## Abbreviations

AIC: Akaike information criteria; FAS: Family affluence scale; GIS: Geographic information system; HBSC: Health Behaviour in School-aged Children; ICC: Intra-class correlation coefficient; OR: Odds ratio; PAR: Population attributable risk; RR: Relative risk.

## Competing interests

The authors declare that they have no competing interests.

## Authors’ contributions

LS was responsible for study design, analysis and composition of the initial draft of the manuscript. IJ and WP helped design the study and provided methodological advice and editorial feedback. AR located the schools using ArcGIS and created the 1 km circular and 1 km road network buffers used in the analysis. All authors read and approved the final manuscript.
